# Potent Natural Soluble Epoxide Hydrolase Inhibitors from *Pentadiplandra brazzeana* Baillon: Synthesis, Quantification, and Measurement of Biological Activities *In Vitro* and *In Vivo*

**DOI:** 10.1371/journal.pone.0117438

**Published:** 2015-02-06

**Authors:** Seiya Kitamura, Christophe Morisseau, Bora Inceoglu, Shizuo G. Kamita, Gina R. De Nicola, Maximilienne Nyegue, Bruce D. Hammock

**Affiliations:** 1 Department of Entomology and Nematology, and University of California Davis Comprehensive Cancer Center, University of California Davis, Davis, California, United States of America; 2 Consiglio per la Ricerca e la sperimentazione in Agricoltura, Centro di Ricerca per le Colture Industriali (CRA-CIN), Bologna, Italy; 3 Départment of Biochemistry and Départment of Microbiology, University of Yaoundé I, Yaoundé, Cameroon; University of Graz, AUSTRIA

## Abstract

We describe here three urea-based soluble epoxide hydrolase (sEH) inhibitors from the root of the plant *Pentadiplandra brazzeana*. The concentration of these ureas in the root was quantified by LC-MS/MS, showing that 1, 3-bis (4-methoxybenzyl) urea (**MMU**) is the most abundant (42.3 μg/g dry root weight). All of the ureas were chemically synthesized, and their inhibitory activity toward recombinant human and recombinant rat sEH was measured. The most potent compound, **MMU**, showed an IC_50_ of 92 nM via fluorescent assay and a *K*i of 54 nM via radioactivity-based assay on human sEH. **MMU** effectively reduced inflammatory pain in a rat nociceptive pain assay. These compounds are among the most potent sEH inhibitors derived from natural sources. Moreover, inhibition of sEH by these compounds may mechanistically explain some of the therapeutic effects of *P. brazzeana*.

## Introduction

Soluble epoxide hydrolase (sEH, EC 3.3.2.10) is the major enzyme responsible for the hydrolysis of epoxy fatty acids (EpFAs) to their corresponding vicinal diols in humans and other mammals [1]. These EpFAs include the epoxides of linoleic, arachidonic, eicosapentaenoic, and docosahexaenoic acid that are produced primarily by cytochrome P450s. These natural molecules are pleiotropic endogenous mediators with key functions in inflammation [1], pain [2], and blood pressure regulation [3]. Increasing the levels of endogenous EpFAs by inhibiting sEH has been shown to block and resolve inflammation [4], reduce pain [2,5,6], and lower blood pressure [7] in several *in vivo* models. Recently, sEH inhibitors were shown to be effective against neuropathic diabetic pain in rodent models [8], and against equine laminitis which is a complex and often fatal disease involving inflammation, hypertension, and severe neuropathic pain [9]. Consequently, sEH has emerged as a potential pharmaceutical target. One sEH inhibitor, AR9281, has undergone a phase II clinical trial for the treatment of hypertension and impaired glucose tolerance [10]. Two other clinical trials are now underway with a different sEH inhibitor that targets chronic obstructive pulmonary disease by Glaxo Smith Kline [11,12]. Several recent studies show that at least some of the beneficial effects associated with dietary supplementation of omega-3 fatty acids (fish oils) are due to the corresponding epoxide metabolites of omega-3 fatty acids [2,13]. Thus sEH inhibitors appear to enhance the positive effects of diet supplementation with fish oils.

A few sEH inhibitors from natural products have been identified. Buscato *et al*. (2013) screened a library of compounds isolated from entomopathogenic bacteria and reported isopropylstilbene as an sEH inhibitor with an IC_50_ of 10 μM [14]. Additionally, Lee *et al*. (2013) reported honokiol and β-amyrin acetate as sEH inhibitors with IC_50_ values of 3.4 μM and 0.57 μM, respectively [15]. However, the potency of these natural compounds is significantly lower than that of synthetic sEH inhibitors which can possess IC_50_ values in the low nM to pM range [16,17]. Although purified natural products have been shown to function as sEH inhibitors *in vitro*, the *in vivo* efficacy of these natural products as sEH inhibitors is yet to be reported.

In our efforts to search for potent sEH inhibitors from natural products and elucidate their possible therapeutic and nutraceutical applications, we focused on the central pharmacophore of known sEH inhibitors with high potency. The 1,3-disubstituted urea is known as a pharmacophore of potent sEH inhibitors [18]. The urea pharmacophore mimics both the epoxide substrate and the transition state of epoxide hydrolysis, leading to competitive inhibition of sEH. Lipophilic substitutions on the urea are favored for improved potency [19].

Tsopmo *et al*. reported the isolation and identification of disubstituted urea compounds in the root of the plant *Pentadiplandra brazzeana* [20]. *Pentadiplandra brazzeana* (commonly known as Oubli in French) is the sole species in the plant genus *Pentadiplandra*. This plant grows either as a shrub or as a liana, and is native to Cameroon and other places in West and Central Africa [21–23]. The root of this plant is used as a folk remedy against hemorrhoids [24], toothache [25], and as an analgesic for the treatment of chest, abdominal, and intercostal pain, as well as rheumatic disorders [23]. Moreover, the essential oil obtained from the root has anti-inflammatory effects [26]. Tsopmo *et al*. have isolated and identified three 1,3-dibenzyl ureas in the root of *P*. *brazzeana* [20]. However, the biological activity of these ureas was not evaluated.

On the basis of structural analogy, we hypothesized that urea compounds in *P*. *brazzeana* are inhibitors of human sEH. To test this hypothesis we measured the inhibitory potency of the crude root extract as well as the individual ureas found in *P*. *brazzeana* against recombinant human and recombinant rat sEH. The amount of these inhibitors was quantified using LC-MS/MS, and the analgesic efficacy of the most potent and abundant compound (**MMU**) was measured in a nociceptive assay using a rat inflammatory pain model.

## Materials and Methods

### General

All reagents and solvents were purchased from commercial suppliers and were used without further purification. Honokiol (purity>98%) was purchased from R&D systems (Minneapolis, MN) and stored at 4°C. All of the synthetic reactions were performed in an inert atmosphere of dry nitrogen or argon. Melting points were determined using an OptiMelt melting point apparatus and are uncorrected. ^1^H and ^13^C-NMR spectra were collected using a Varian 600 MHz spectrometer with chemical shifts reported relative to residual deuterated solvent peaks or tetramethylsilane internal standard. Accurate masses were measured using a Micromass LCT ESI-TOF-MS. FT-IR spectra were recorded on a Thermo Scientific NICOLET IR100 FT-IR Spectrometer.

### Ethics Statement

The plant samples were harvested under the authority of National Herbarium of Cameroon by Mrs. Ada, a Cameroonian botanist at the National Herbarium of Cameroon. The National Herbarium of Cameroon is the authority in charge of the promotion of research on plants. Cameroonian researchers do not need permission to collect plant samples in Cameroon.

For the nociceptive assays, all of the studies were conducted in line with U.S. federal government regulations and were approved by the Institutional Animal Care and Use Committee at the University of California, Davis.

### Plant material and sample preparation

The plant root samples were harvested on February 5^th^ 2010 at Elounden (Yaoundé, Cameroon) by Mrs. Ada, a botanist at the National Herbarium of Cameroon in Yaoundé. A voucher specimen is kept at the National Herbarium of Cameroon in Yaoundé (Identication No. 6538NM/01). Root material was freeze-dried, reduced to a fine powder and kept at -20°C until used for the analysis.

### DNA extraction and sequencing of ribosomal DNA and maturase K DNA partial sequences

Total DNA in the root powder was extracted using a Qiagen DNeasy Plant Mini Kit (Qiagen, Valencia, CA, USA) following the manufacturer’s protocol. The partial sequences of 18S ribosomal DNA and maturase K DNA were amplified by PCR. The sequences of the PCR primers are as follows: 18S ribosomal DNA, forward primer-1 (5′-GCCGCGGTAATTCCAGCTCCAATAGCGTATATTT-3′) and reverse primer-1 (5′-GAGTCCTAAAAGCAACATCCGCTGATCCCTG-3′); and forward primer-2 (5′-GCAGTTAAAAAGCTCGTAGTTGGACCTTGGGATG-3′) and reverse primer-2 (5′-TGAGACTAGGACGGTATCTGATCGTCTTCGAG-3′). Maturase K DNA, forward primer-1 (5′-GGAGGAATTTCAAGTATATTTAGAGTTGGATAGAGTTCGGC-3′) and reverse primer-1 (5′-CGCAAGAAATGCAAAGAAGAGGCATCTTTTACCCTG-3′); and forward primer-2 (5′-GCAGTTAAAAAGCTCGTAGTTGGACCTTGGGATG-3′) and reverse primer-2 (5′- TGAGACTAGGACGGTATCTGATCGTCTTCGAG-3′). PCR amplification was performed with these primers using GoTaq Green Master Mix (Promega, Madison, WI, USA) as follows: 95°C, 2 min; 35 cycles of 95°C, 30 sec; 62°C, 30 sec; and 72°C, 45 sec; followed by 72°C, 5 min. This PCR generated a 0.5 kbp-long amplicon that was column-purified using a QIAquick Gel Extraction Kit (Qiagen, Valencia, CA, USA) following the manufacturer’s protocol. The sequences of PCR products were determined by the UC Davis College of Biological Sciences Sequencing Facility.

### Extraction

The dried root powder (100 mg) was extracted with dichloromethane (DCM)-methanol (MeOH) (1:1) (3 x 2 ml) at room temperature (24 h per extraction). Concentration of the combined percolates *in vacuo* yielded a yellowish crude extract (approximately 8 mg).

### Synthesis of ureas

**Fig 1:**
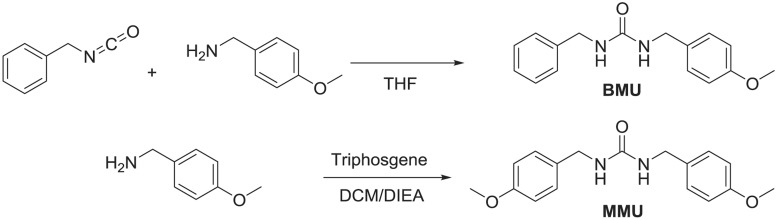
Synthetic schemes of urea compounds found in *P*. *brazzeana*. THF = tetrahydrofuran, DCM = dichloromethane, DIEA = diisopropylethylamine.

1,3-Dibenzylurea (**BBU**) was previously synthesized [27]. Fig. 1 shows the synthetic scheme for the other 2 ureas described in this study.

1-Benzyl-3-(4-methoxybenzyl) urea (**BMU**)

To a solution of benzylisocyanate (158 μl, 1.28 mmol) in THF (2 ml) was added 167 μl (1.28 mmol) of (4-methoxyphenyl) methanamine. After stirring for 10 min at room temperature, hexane was added and the resulting white crystals were collected (260 mg, 0.963 mmol, 75%). mp 139.6–140.2°C; ^1^H-NMR (DMSO-*d*_6_, 600 MHz); δ (ppm) 7.31 (2H, dd, *J*_*1*_ *=* 7.5 Hz, *J*_*2*_ *=* 5.0 Hz) 7.25–7.20 (m, 3H), 7.17 (2H, d, *J =* 8.4 Hz), 6.87 (2H, d, *J =* 8.4 Hz), 6.38 (1H, t, *J* = 6.0 Hz), 6.33 (1H, t, *J* = 6.0 Hz), 4.22 (2H, d, *J* = 6.0 Hz), 4.15 (2H, d, *J* = 6.0 Hz), 3.73 (3H, s), ^13^C NMR (151 MHz, DMSO-*d*_6_): δ 158.1, 158.0, 140.9, 132.8, 128.3, 128.2, 127.0, 126.5, 113.6, 55.0, 42.9, 42.4; IR (cm^-1^) 3349, 3317, 3032, 2923, 1625, 1577, 1511, 1242, 1031, ESI-MS [M+Na]^+^ m/z 293.11 (calcd for C_16_H_18_N_2_NaO_2_ 293.13).

1, 3-Bis (4-methoxybenzyl) urea (**MMU**)

To an ice-cold solution of triphosgene (100 mg, 0.34 mmol) in 2 ml DCM was added dropwise a solution of 4-methoxybenzylamine (137 mg, 1 mmol) and *N*,*N*,-diisopropylethylamine (DIEA) (191 μl, 1.1 mmol) in 2 ml of DCM. This mixture was stirred for 5 min at 0°C. To this mixture was added 4-methoxybenzylamine (137 mg, 1 mmol) and DIEA (191 μl, 1.1 mmol) in 2 ml of DCM, warmed slowly to room temperature, and stirred overnight. To this solution was added DCM, and washed with 1 M aqueous HCl solution three times. The DCM layer was dried over MgSO_4_ and concentrated. Recrystallization from the DCM resulted in 45 mg (0.15 mmol, 15%) of target compound as a white powder; mp 177.7–178.4°C; ^1^H-NMR (DMSO-*d*_6_, 600 MHz); δ (ppm) 7.16 (4H, d, *J* = 9.0 Hz), 6.87 (4H, d, *J =* 8.4), 6.27 (2H, t, *J =* 6.0), 4.14 (4H, d, *J =* 6.0), 3.72 (6H, s), ^13^C NMR (151 MHz, DMSO-*d*_6_): δ 158.0, 158.0, 132.8, 128.3, 113.6, 55.0, 42.4; IR (cm^-1^) 3350, 3318, 2955, 2924, 2837, 1624, 1577, 1509, 1237, 808, ESI-MS [M+Na]^+^ m/z 323.13 (calcd for C_17_H_20_N_2_NaO_3_ 323.14).

### LC-MS/MS analysis

Urea compounds were quantified using a Waters Quattro Premier triple quadrupole tandem mass spectrometer (Micromass, Manchester, UK) interfaced to an electrospray ionization (ESI) source. The ESI was performed following HPLC in the positive mode at 2.51 kV capillary voltage. The source and the desolvation temperatures were set at 120 and 300°C, respectively. Cone gas (N_2_) and desolvation gas (N_2_) were maintained at ﬂow rates of 10 and 700 l/h, respectively. Optimized conditions for mass spectrometry are shown in Table 1. Dwell time was set to 0.1 s. A regression curve for each compound was obtained from at least eight different concentrations of standard stock solutions (r^2^ > 0.99). 1, 3-Diphenylurea (**DPU**) was used as an internal standard and was added just before the analysis. The final concentration of 1, 3-diphenylurea was adjusted to 100 nM. Full scan MS/MS spectra (*m/z* interval of 10–300) were collected for each of the molecules (precursor ion; **MMU** *m/z*: 301, **BMU** *m/z*: 271, **BBU** *m/z*: 241) using product ion scan mode with cone voltage and collision voltage of 35V and 26V, respectively. The MS was coupled with a Waters Acquity UPLC (Waters, and Milford, MA, USA). The HPLC column Imtakt Cadenza CD-C18 (CD007, 3 μm, 4.6x500 mm) was used to separate these analytes. The HPLC solvent gradient is shown in Table 2. The infusion flow rate to the MS was adjusted to 0.3 ml/min.

**Table 1:** Optimum mass transition conditions and key fragmentation of the urea compounds.

	Ionization mode		Transition		Cone voltage (V)	Collision voltage (V)
MMU	+	301.2	->	120.9	35	26
BMU	+	271.3	->	120.9	33	23
BBU	+	241.2	->	90.9	30	20
DPU	+	213.1	->	93.9	35	16

**Table 2:** HPLC solvent gradient for the separation of target ureas.

Time ^a^	Aqueous phase ^b^	Organic phase ^c^
0.00	50	50
25.00	32	68
25.01	0	100
35.00	0	100
35.01	50	50
40.00	50	50

^a^ 0.6 ml/min flow rate,

^b^ Milli-Q Water 99.9, Acetic acid 0.1, volume %,

^c^ Acetonitrile 99.9, Acetic acid 0.1, volume %.

### Standard addition and recovery experiments

A solution of **BBU** (20 nmol), **BMU** (100 nmol), and **MMU** (200 nmol) in 10 μl of methanol was spiked into 100 mg of dried root powder. The powder was mixed, dried, and kept at -20°C until extraction. Samples were extracted as described above. The recovery percentage was calculated as follows:

% recovery=(amount in spiked sample-amount in control) × 100 / amount spiked

### Enzyme Purification

Recombinant human and recombinant rat sEH were produced in insect High Five cells using recombinant baculovirus expression vectors, and purified by affinity chromatography as reported previously [28,29]. Each enzyme appeared as a single band (0.3 μg loading) with an estimated purity of more than 95% by Coomassie Brilliant Blue staining following SDS-PAGE separation (S1 Fig.). The final recombinant sEH preparations had no esterase or glutathione *S*-transferase activity which interferes with the CMNPC assay as described below.

### Measurement of sEH inhibition by fluorescent assay (CMNPC assay)

IC_50_ values were determined as described previously [30] using cyano (2-methoxynaphthalen-6-yl) methyl *trans*-(3-phenyl-oxyran-2-yl) methyl carbonate (CMNPC) as a fluorescent substrate. Recombinant human or recombinant rat sEH (0.96 nM) was incubated with crude root extract or inhibitors for 5 min in 25 mM bis-Tris/HCl buffer (pH 7.0) containing 0.1 mg/mL of BSA at 30°C prior to substrate introduction ([S] = 5 μM). Activity was measured by determining the appearance of the 6-methoxy-2-naphthaldehyde with an excitation wavelength of 330 nm and an emission wavelength of 465 nm for 10 min.

### Measurement of sEH inhibition by radioactivity-based assay (*t*-DPPO assay)

IC_50_ values were determined as described previously [31] with slight modifications, using racemic [^3^H] *trans*-diphenylpropene oxide (*t*-DPPO). Purified recombinant human sEH was diluted in 100 mM sodium phosphate buffer (pH 7.4) containing 0.1 mg/ml BSA, and was incubated in triplicate with inhibitors for 5 min at 30°C prior to the introduction of the radiolabeled substrate (*t-*DPPO: 50 μM; ∼10,000 cpm/assay). The mixture was incubated at 30°C for 10 min and the reaction was quenched by the addition of 60 μl of methanol. The remaining substrate was extracted by vigorous mixing with 200 μl of isooctane. The radioactivity of the aqueous phase was measured using a liquid scintillation counter (Perkin Elmer Tri-Carb 2810TR, Shelton, CT). Epoxide hydrolase activity was determined as a percentage of the radioactivity corresponding to the diol in the aqueous phase relative to the control.

### Enzyme kinetics

Dissociation constants were determined following the method described by Dixon [32] for competitive tight binding inhibitors, using *t*-DPPO as a substrate [31]. Inhibitor **MMU** at concentrations between 0 and 1000 nM was incubated in triplicate for 5 min in 100 mM sodium phosphate buffer (pH 7.4) at 30°C with 100 μl of the enzyme (2 nM of recombinant human sEH). Substrate (4.0 ≤ [S] _final_ ≤ 30 μM) was then added. Velocity was measured as described previously [31]. For each substrate concentration, the plots of the velocity as a function of the inhibitor concentration allow the determination of an apparent inhibition constant (*K*iapp [32]). The plot of the *K*iapp values as a function of the substrate concentration allows the determination of *K*i when [S] = 0. *K*iapp was measured in at least 3 separate assays. Results are presented as average ± SE.

### Measurement of solubility of MMU

An excess amount of **MMU** was added to a vial containing sodium phosphate buffer, 0.1 M pH 7.4 (0.25 mL), and the **MMU** suspension was equilibrated during 1 h of sonication and 48 h of shaking at 25°C, followed by centrifugation. The amount of **MMU** in the supernatant was analyzed by LC—MS/MS. The result is presented as mean ± SD of triplicate measurement.

### Nociceptive assay using rat inflammatory Pain Model

Male Sprague-Dawley rats weighing 235–280 g were obtained from Charles River Laboratories and maintained in the UC Davis animal housing facility with ad libitum water and food on a 12 h/12 h light-dark cycle. Behavioral nociceptive testing was conducted by assessing mechanical withdrawal threshold using an electronic von Frey anesthesiometer apparatus (IITC, Woodland Hills, CA) [33]. The controller was set to “maximum holding” mode so that the highest applied force (in grams) upon withdrawal of the paw was displayed. At least three measurements were taken at 1–2 min inter-stimulus intervals. Data were normalized to percentage values using the formula (mechanical withdrawal threshold (g)) ×100/ (mechanical withdrawal threshold (g) of naïve animals). The analgesic effect of **MMU** was tested using the intraplantar carrageenan elicited local inflammatory pain model [16,33]. Following baseline measurements, carrageenan (50 μl, 1% solution of carrageenan) was administered into the plantar area of one hind paw in order to induce inflammatory pain. At 4 h post administration of carrageenan, postcarrageenan responses were measured, and immediately afterwards, **MMU** (10 μg in 10 μl of PEG400), morphine sulfate (10 μg in 10 μl saline), the potent synthetic sEH inhibitor 1-(1-(Cyclopropanecarbonyl) piperidin-4-yl)-3-(4-(trifluoromethoxy) phenyl) urea (**TPCU**) (10 ng in 10 μl of PEG400), or the vehicle (10 μl of PEG400) was administered into the inflamed paw by intraplantar injection. Subsequently, the ability of these treatments to reduce the carrageenan-induced inflammatory pain was monitored over the course of 2.5 h.

### Statistical Analysis

Data were analyzed using SigmaPlot 11.0 for windows (Systat Software Inc., San Jose, CA). Kruskal-Wallis One Way ANOVA on Ranks followed by Tukey Test was performed with p values<0.05 considered signicant.

## Results and Discussion

### Inhibition of human soluble epoxide hydrolase by crude root extract

Determination of the plant species of the root tissues was supported by comparing the partial sequences of 18S ribosomal DNA (NCBI accession number: AF070972) and maturase K DNA (AY483239) as described in a previous study [34]. The sequence alignments are shown in S1 Text. The crude root extract of *P*. *brazzeana* was prepared by percolation in DCM-methanol (1:1), and the inhibitory activity of the extract toward human sEH was measured by both fluorescent assay (CMNPC assay) and radioactivity based assay (*t*-DPPO assay) [30,31]. The root extract showed an IC_50_ value of 1.9 ± 0.4 μg crude extract/ml in the CMNPC assay and 35 ± 3 μg crude extract/ml in the *t-*DPPO assay.

### Optimization of LC-MS/MS and analysis of plant extracts

**Fig 2:**
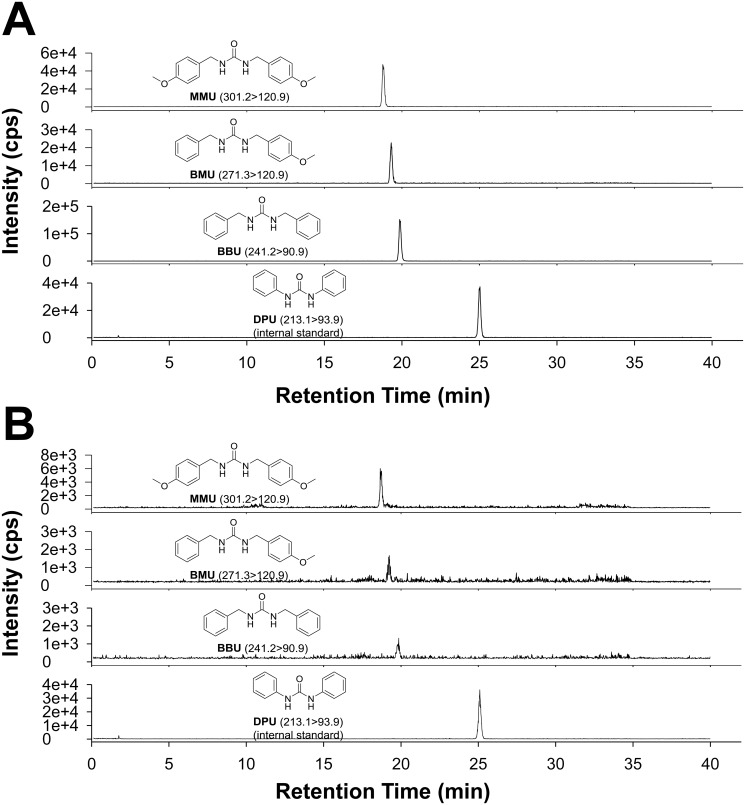
Root of P. brazzeana contains urea-based sEH inhibitors. The chemical structure of target analytes and their mass transition are shown as inserts in the figure. The LC-MS/MS conditions are described in Materials and Methods. (A) MRM chromatograms of synthetic standard ureas. (B) MRM chromatograms of crude root extract from *P*. *brazzeana* (250 μg root/ml, 10μl). cps: counts per second.

Two of 3 urea derivatives found in *P*. *brazzeana* as reported by Tsopmo *et al*. [20] were synthetically prepared as shown in Fig. 1. Fig. 2A shows a representative chromatogram of the separation of synthetic standards of the 3 ureas reported by Tsopmo *et al*. with our internal standard, 1,3-diphenylurea (**DPU**). The LC-MS/MS analysis clearly showed that the plant root extract contains these ureas, based on the retention time of the analytes and the mass to charge ratio of the molecular and fragment ions (Figs. 2B and S2–S4).

To determine the concentration of these ureas in plant samples an LC-MS/MS method was optimized in multiple reaction monitoring (MRM) mode. The concentrations of these ureas are shown in Table 3. 1, 3-bis (4-Methoxybenzyl) urea (**MMU**) was the most abundant, followed by an asymmetric urea (**BMU**) and then non-substituted benzyl urea (**BBU**). In order to determine the extraction efficiency from the plant sample, standard addition and recovery experiments were performed. As shown in Table 3, the recovery percentages of **MMU**, **BMU**, and **BBU** were between 89–99%, showing that our method efficiently extracted the target analytes.

**Table 3:** Concentration of ureas in the root of *P*. *brazzeana* and extraction recovery percentage.

	Concentration	Recovery
	(μg/ g dry root weight)^1^	(%)^2^
MMU	42.3±4.2	94±2.8
BMU	13.7±1.5	89±2.9
BBU	1.9±0.2	99±5.4

^1^ n = 4, mean ± standard deviation are shown.

^2^ n = 5, mean ± standard deviation are shown.

### sEH inhibitory activity of ureas from *P*. *brazzeana*

The ability of **MMU**, **BMU**, and **BBU** to inhibit the activity of human sEH was measured using two different substrates. The IC_50_ values of these urea compounds are shown in Table 4. As reported previously, the *t*-DPPO assay gives higher IC_50_ values (lower potency) for the same compound than the CMNPC assay [30]. The symmetric bis (methoxybenzyl) urea (**MMU**), which was found at the highest concentration in the root extract, showed the highest potency toward human sEH when determined with the CMNPC or *t-*DPPO assays. The inhibitory activity of the asymmetric urea (**BMU**) was of about 4-fold lower than that of **MMU** with both assays. The non-substituted benzyl urea, **BBU**, showed the lowest potency. These results are consistent with the previous structure-activity relationship of sEH inhibitors in that *para* methoxy substitution on the phenyl ring leads to higher potency in comparison to non-substituted inhibitors [16].

**Table 4:** sEH inhibition potency of ureas found in the root of *P*. *brazzeana*.

	Human	Human	Rat
	sEH IC_50_ (nM)	sEH IC_50_ (μM)	sEH IC_50_ (nM)
	(CMNPC assay)^1^	(*t*-DPPO assay)^1^	(CMNPC assay)^1^
MMU	92±14	2.6±0.4	35±5
BMU	400±50	10.2±2.6	44±4.2
BBU	1900±300	48.3±7.0	100±6
honokiol	ND^2^	20.3±1.7	ND^2^

^1^ Measured three times and mean ± standard deviation are shown.

^2^ ND: Not determined.

In order to compare our compounds with previously reported natural product sEH inhibitors, the potency of honokiol was measured using our radioactivity-based assay. Honokiol showed an IC_50_ of 20.3 μM with the *t*-DPPO assay. Based on this result, **MMU** is about 8-fold more potent than honokiol.

The inhibitory potency of these compounds against affinity purified, recombinant rat sEH was measured using the CMNPC assay. The ureas from *P*. *brazzeana* showed a similar pattern of inhibitory potency with the rat recombinant sEH as was found with the recombinant human sEH (Table 4).

### Enzyme inhibition kinetics

**Fig 3:**
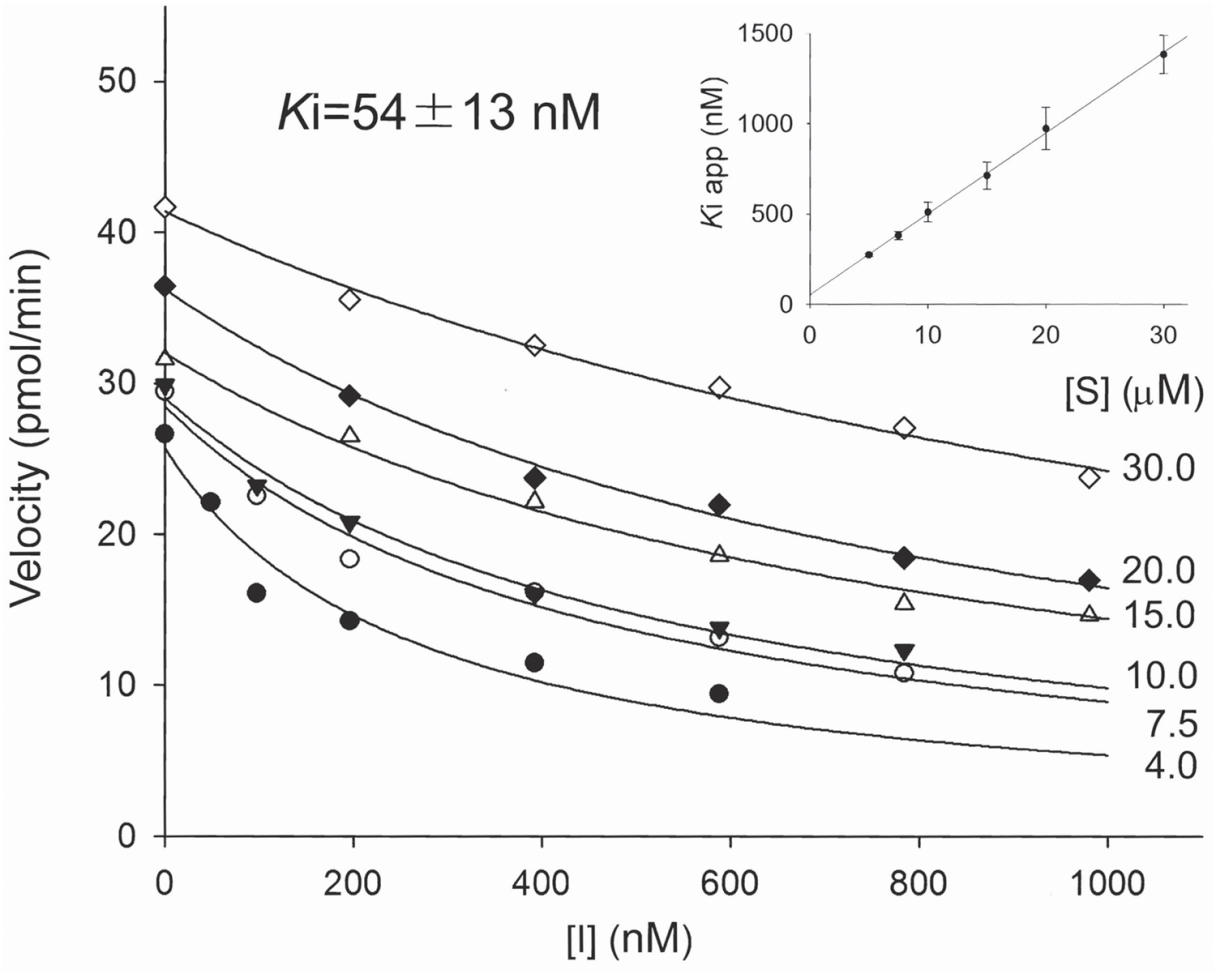
Enzyme inhibition kinetics of MMU on human soluble epoxide hydrolase. Enzyme kinetic analysis was performed on recombinant human soluble epoxide hydrolase (2 nM) using [^3^H] *t*-DPPO as a substrate. For each substrate concentration (4.0–30.0 μM), the velocity was plotted as a function of inhibitor concentration (0–1000 nM), allowing the determination of an apparent inhibition constant (*K*iapp). *K*iapp values are plotted as a function of the substrate concentration (insert). For [S] = 0, a *K*i value of 54 nM was found.

In order to define the mode of enzyme inhibition and to determine the dissociation constant (*K*i), we performed enzyme kinetic analysis of **MMU** using recombinant human sEH. As shown in the insert in Fig. 3, the *K*iapp values increase with an increase in the concentration of the substrate. This strongly suggests that **MMU** inhibits sEH as a competitive inhibitor, which is consistent with other urea-based sEH inhibitors [18]. The dissociation constant (*K*i) of **MMU** was determined to be 54±13 nM. In terms of potency this is approximately 40-fold lower than the highly potent synthetic inhibitor *t*-AUCB (*K*i = 1.5 nM), but approximately 2-fold higher than the synthetic sEH inhibitor APAU (*K*i = 125 nM) which has undergone a phase II clinical trial [35,36].

### Nociceptive assay in rat inflammatory pain model

**Fig 4:**
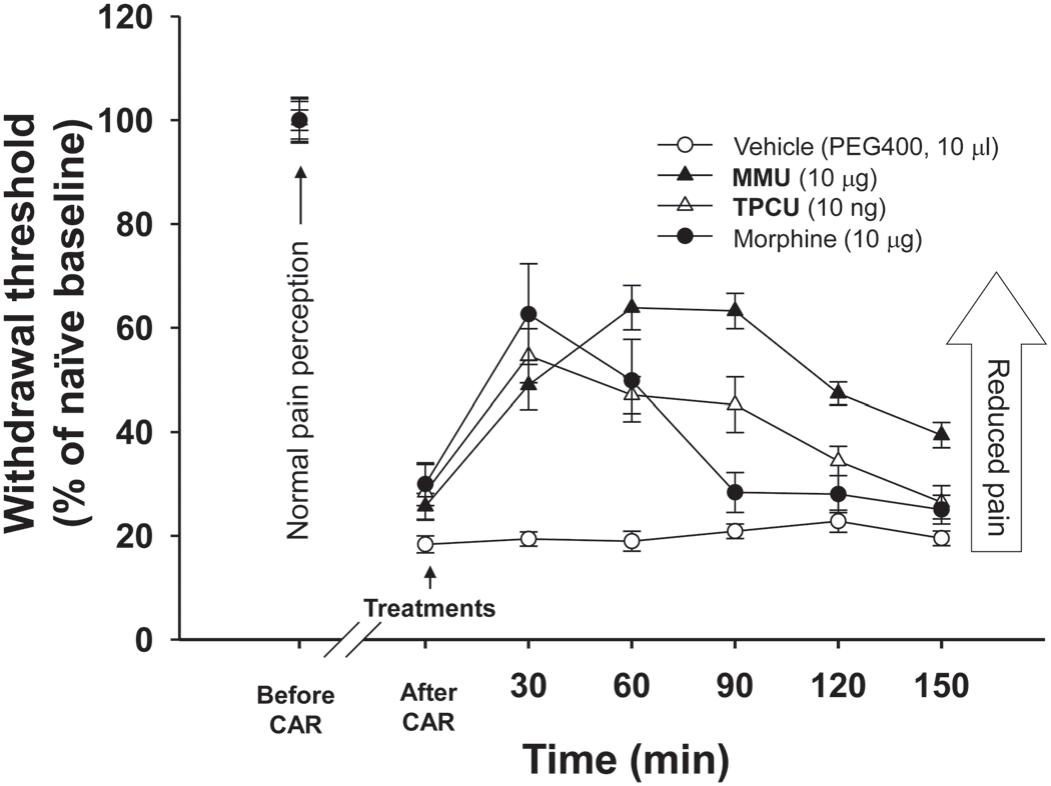
MMU effectively reduces carrageenan-induced inflammatory pain in rat. Administration of the inflammatory agent carrageenan (CAR) induces a stable hyperalgesic response for the duration of the experiment. Treatment with **MMU** (▲, 10 μg/paw, intraplantar), morphine (●, 10 μg/paw, intraplantar) or the potent synthetic sEH inhibitor (**TPCU**) (△, 10 ng/paw, intraplantar) significantly reduced pain levels (Kruskal-Wallis One Way ANOVA on Ranks, p≤0.001, Tukey’s post hoc test (**MMU** vs vehicle, morphine vs vehicle, **TPCU** vs vehicle, and **MMU** vs morphine) p<0.05). Mean ± SE (n = 6) of mechanical withdrawal threshold (% of naïve baseline) are shown. Vehicle (○), morphine (●), and **TPCU** (△) treatment data are from Rose *et al*., (2010).

Several studies have shown that sEH inhibitors effectively reduce inflammatory pain [4,5,16]. In order to evaluate the *in vivo* efficacy of **MMU**, a rat inflammatory pain model was used to measure its analgesic effects. **MMU** was selected based on its inhibitory potency on sEH and its concentration in *P*. *brazzeana*. As shown in Fig. 4, local administration of either **MMU** (10 μg/intraplantar injection), morphine (10 μg/intraplantar injection), or potent synthetic sEH inhibitor **TPCU** (10 ng/intraplantar injection) significantly reduced carrageenan induced inflammatory pain (Kruskal-Wallis One Way ANOVA on Ranks, p≤0.001). In this assay, **MMU**, **TPCU**, and morphine appeared to have similar efficacy in terms of reducing enhanced pain perception but **MMU** showed a longer duration of efficacy compared to morphine (**MMU** vs morphine, Tukey’s post hoc test, p<0.05). **TPCU** showed an IC_50_ of 0.4 nM with *K*i value of 0.67 nM on human sEH [16,36], which is approximately 230-fold more potent than **MMU** based on the IC_50_ values. Thus, it is reasonable that **MMU** showed similar or slightly better efficacy in comparison to **TPCU** when **MMU** was administered at a 1000-fold higher dose than **TPCU**.

The root of *P*. *brazzeana* is used as a traditional analgesic against various types of pain [23,25]. For example, dried root bark powder is applied by scarification to treat intercostal and abdominal pain, while the crushed root or root bark is applied as a topical ointment or as an infusion drink to soothe chest pain, lumbago, rheumatism, and haemorrhoids [23]. In order to relate our results to the ethnomedicinal use of *P*. *brazzeana*, it is necessary to determine how much of the ureas are absorbed into the body, and whether they reach a dose that can effectively inhibit sEH. In the case of application by scarification, which is similar to intraplantar injection, these urea compounds could be absorbed and reach the site of action. In the case of oral administration, **MMU** may have limited bioavailability given that the synthetic **MMU** has low solubility (10.6±2.1 μM (mean±SD)) in sodium phosphate buffer (pH7.4). It should be noted, however, that the root of *P*. *brazzeana* may have compounds such as lipids and polysaccharides, which could solubilize **MMU**, **BMU** or **BBU** and facilitate their absorption after ingestion. Determination of the bioavailability of these inhibitors after oral administration of the root, or topical ointment treatment is the subject of further research. A recent study showed that sEH inhibitors are highly effective against neuropathic diabetic pain [8]. Determining the efficacy of the compound/plant extract on neuropathic pain is also the subject of further study. Determining the localization of **MMU** within the plant root, especially whether it is localized within the root bark, will help us to optimize a method for efficient isolation of **MMU** or other target ureas from *P*. *brazzeana*.

The theoretical IC_50_ of the crude extract of *P*. *brazzeana* against recombinant human sEH is approximately 29 μg crude root extract/ml using the CMNPC assay. This calculated IC_50_ of the crude root extract is based on the IC_50_ of **MMU** and its concentration in the plant (assuming for simplicity that **MMU** is the main component that inhibits sEH). Experimentally, however, the crude root extract gave an IC_50_ of 1.9 μg crude root extract/ml. This experimentally-derived potency is approximately 15-fold higher than the calculated potency, suggesting that the crude root extract contains unknown sEH inhibitor(s), or another factor that improves its potency. It should be noted, however, that in different preparations of the crude root extract, significant variation in the inhibitory potency (IC_50_ = 4.1–176 μg/ml in *t*-DPPO assay) was found while the concentrations of **MMU**, **BMU**, and **BBU** were very similar. This may indicate that the root extract contains unknown inhibitors that are sensitive to the extraction process that is utilized. Optimization of the extraction/processing method is still needed in order to precisely measure the level of sEH inhibition by the crude plant extract. In our preliminary HPLC separation of the crude root extract and measurement of sEH inhibition by each fraction, we observed strong sEH inhibition in fractions that were different from the fractions containing **MMU** (S5 and S7 Figs., S1 and S2 Tables). This indicates that there are other unidentified sEH inhibitors in this plant. Addition of dithiothreitol (100 μM) to either the crude extract or to each fraction did not change the inhibitory potency, indicating that the unknown inhibitors are not Michael acceptors. The purification and identification of these inhibitors are the subjects of further study.

**MMU** and **BMU** have not been reported in other plants, while **BBU** has been detected in plants such as moringa (*Moringa oleifera*) and garden cress (*Lepidium sativum*) [37,38]. A regioisomer of **MMU**, bis (*m-*methoxybenzyl) urea (salvadourea), was reported in the miswak, *Salvadora persica*, plant [39]. Interestingly, *P*. *brazzeana*, moringa, garden cress, and miswak all belong to the plant order Brassicales. In addition these plants produce glucosinolates or their corresponding isothiocyanate products [40–43], suggesting that they may share a metabolic pathway. A genetic comparison between these species based on ribosomal DNA sequences, however, failed to find any similarity between these species. Chemotaxonomical analysis is needed to find shared biosynthetic pathways in these plants.

## Conclusions

We demonstrate that urea compounds found in the root of *P*. *brazzeana* have potent sEH inhibitory activities. These inhibitors were quantified by LC-MS/MS, showing that the most potent inhibitor, **MMU**, is found at the highest concentration in the plant root in comparison to **BMU** and **BBU**. **MMU** showed local analgesic effects in an *in vivo* inflammatory pain model. These findings may explain, at least in part, the pharmacological mechanisms of traditional medicinal uses of the root of *P*. *brazzeana*. Our findings illustrate that plants could provide leads for novel and therapeutically valuable sEH inhibitors and that plant natural products that inhibit sEH could be valuable supplements to an omega-3 fatty acid-rich diet.

## Supporting Information

S1 Fig SDS-PAGE analysis of human and rat recombinant sEH for purity assessment. Each enzyme appeared as a single band (0.3 μg loading) of ca. 62 kDa by Coomassie Brilliant Blue staining following SDS-PAGE separation. The migration of molecular weight markers (in kDa) are indicated to the left.
(TIF)

S2 Fig Full scan MS/MS spectra of MMU synthetic standard (upper panel) and plant extract (lower panel). Full scan MS/MS spectra (*m/z* interval of 10–300) were collected for **MMU** setting precursor ion *m/z* as 301 with cone voltage and collision voltage of 35V and 26V, respectively. cps: counts per second.
(TIF)

S3 Fig Full scan MS/MS spectra of BMU synthetic standard (upper panel) and plant extract (lower panel). Full scan MS/MS spectra (*m/z* interval of 10–300) were collected for **BMU** setting precursor ion *m/z* as 271 with cone voltage and collision voltage of 35V and 26V, respectively. cps: counts per second.
(TIF)

S4 Fig Full scan MS/MS spectra of BBU synthetic standard (upper panel) and plant extract (lower panel). Full scan MS/MS spectra (*m/z* interval of 10–300) were collected for **BBU** setting precursor ion *m/z* as 241 with cone voltage and collision voltage of 35V and 26V, respectively. cps: counts per second.
(TIF)

S5 Fig Normal phase HPLC fraction collection and sEH inhibition by the fractions. Crude root extract (approximately 2 mg) was injected into a normal phase HPLC column (YMC-Pack SIL-06) and eluted with 20% isopropanol in hexane with a flow rate of 4 ml/min for 15 min, followed by 100% isopropanol with a flow rate of 2 ml/min for 25 min. The relative intensity of the UV absorption at the wavelength of 210 and 276 nm are shown (top and middle). Fractions were collected for every 4 ml of eluent. After the solvent was evaporated the residue was reconstituted in 50 μl DMSO. The inhibition percentage by each fractions (100 times dilution of reconstituted solution) was measured using the CMNPC assay with recombinant human sEH (bottom). The black circles (●) represent the inhibition percentage by each of the fractions. The crude extract mixture (100 times dilution of 2 mg extract/ml DMSO) showed complete inhibition of sEH activity (black circle shown at time 0 min). The retention times of the 3 synthetic ureas (**BBU**, **BMU**, and **MMU**) are indicated by arrows in the figure.
(TIF)

S6 Fig Procedure for the reverse phase HPLC fraction collection. (TIF)

S7 Fig Reverse phase HPLC fraction collection and sEH inhibition by the fractions. Crude root extract (approximately 5 mg) was injected into a reverse phase HPLC column (Waters SunFire Prep C18, 5 μm, 10x100 mm) and eluted with 10% acetonitrile in water with a flow rate of 2 ml/min for 10 min, followed by a linear gradient elution of acetonitrile 10% to 100% at a flow rate of 2 ml/min for 25 min, and eluted with 100% acetonitrile for 15 min at a flow rate of 2 ml/min. The relative intensity of the UV absorption at the wavelength of 210 and 280 nm are shown (top and middle). The retention times of the 3 synthetic ureas (**BBU**, **BMU**, and **MMU**) are indicated by an arrow in the figure. Fractions were collected for every 4 ml of eluent. After the solvent was evaporated the residue was reconstituted in 50 μl DMSO. The inhibition percentage by each fractions (100 times dilution of reconstituted solution) was measured using the CMNPC assay with recombinant human sEH (bottom). The black circles (●) represent the inhibition percentage by each of the fractions. The crude extract **C** (100 times dilution of 5 mg extract/ml DMSO) showed complete inhibition of sEH activity (black circle shown at time 0 min).
(TIF)

S1 Table Relative potency of normal phase HPLC fractions. (DOCX)

S2 Table Relative potency of reverse phase HPLC fractions. (DOCX)

S3 Table Effect of extraction solvent on human sEH inhibitory potency. (DOCX)

S1 Text Sequence alignments of PCR products from root sample of P. brazzeana vs sequences in NCBI database. (DOCX)

S2 Text Methods for HPLC fraction collection and sEH inhibition by the fractions. (DOCX)
