# Supplementary figures and images for: Potent Natural Soluble Epoxide Hydrolase Inhibitors from Pentadiplandra brazzeana Baillon: Synthesis, Quantification, and Measurement of Biological Activities In Vitro and In Vivo
Source: PLoS One. 2015 Feb 6;10(2):e0117438. doi: 10.1371/journal.pone.0117438 (PMC4319826; doi:10.1371/journal.pone.0117438)

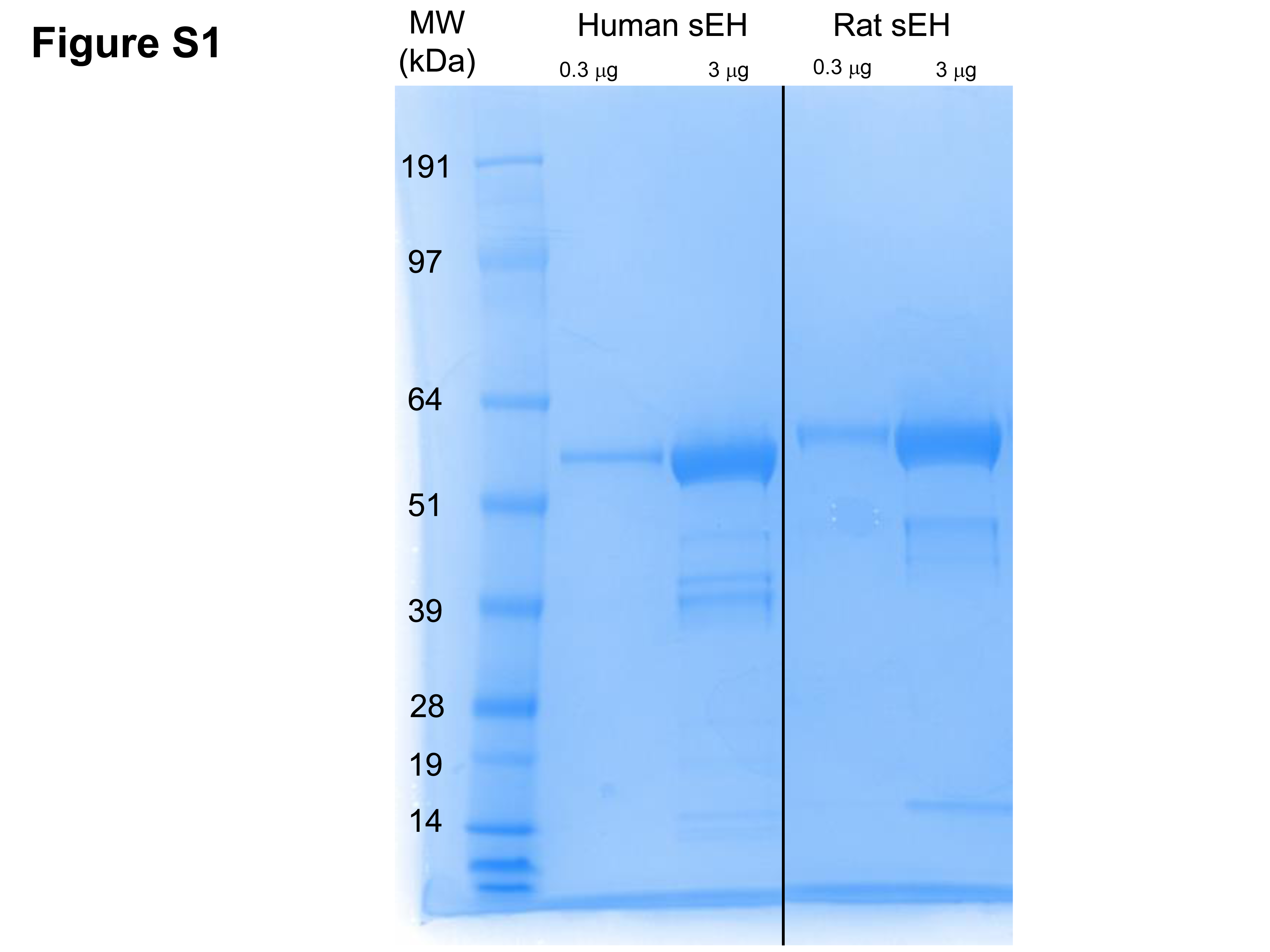

Supplement: S1 Fig — Each enzyme appeared as a single band (0.3 μg loading) of ca. 62 kDa by Coomassie Brilliant Blue staining following SDS-PAGE separation. The migration of molecular weight markers (in kDa) are indicated to the left. (TIF) [file pone.0117438.s001.tif]

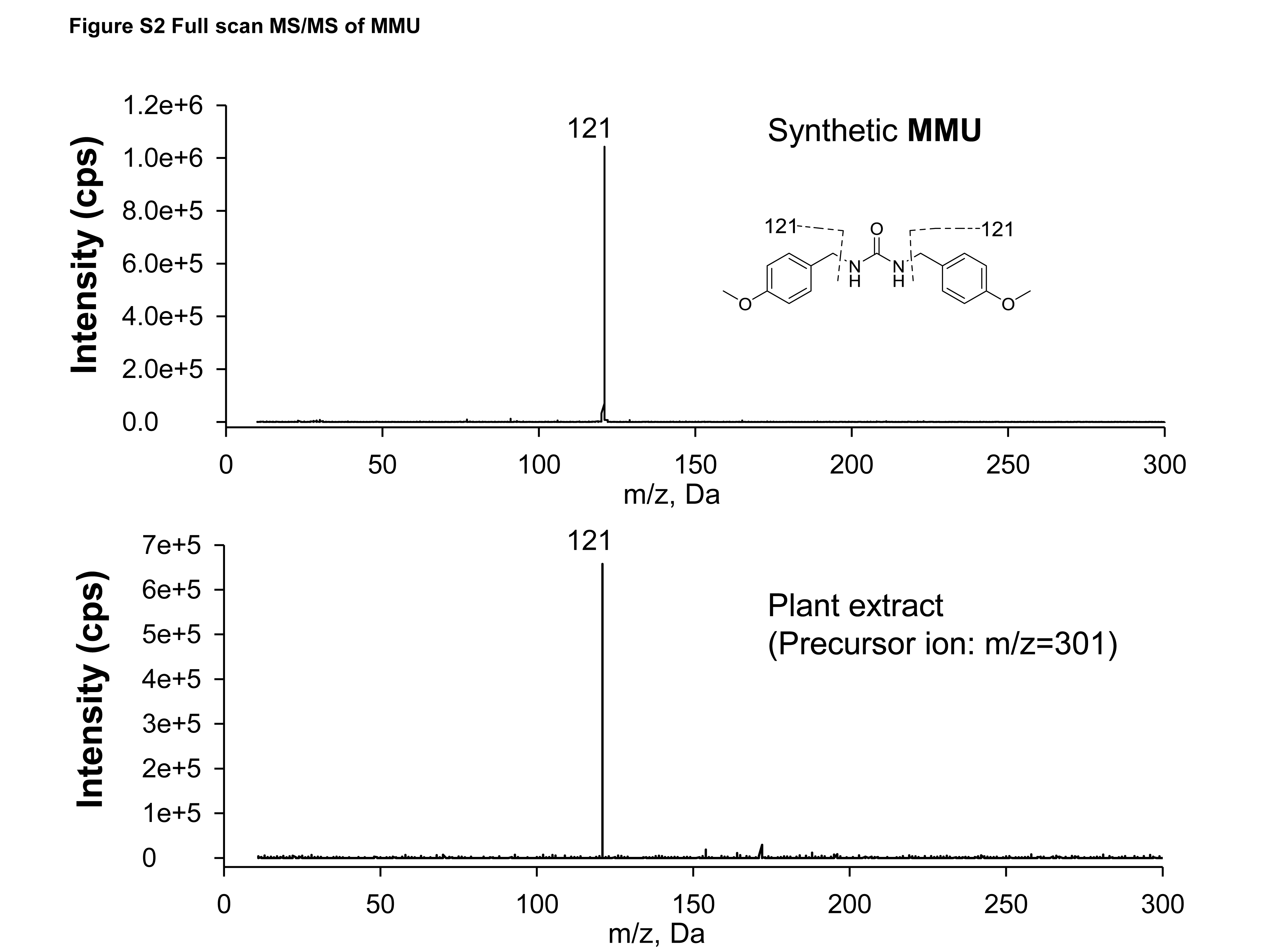

Supplement: S2 Fig — Full scan MS/MS spectra (m/z interval of 10–300) were collected for MMU setting precursor ion m/z as 301 with cone voltage and collision voltage of 35V and 26V, respectively. cps: counts per second. (TIF) [file pone.0117438.s002.tif]

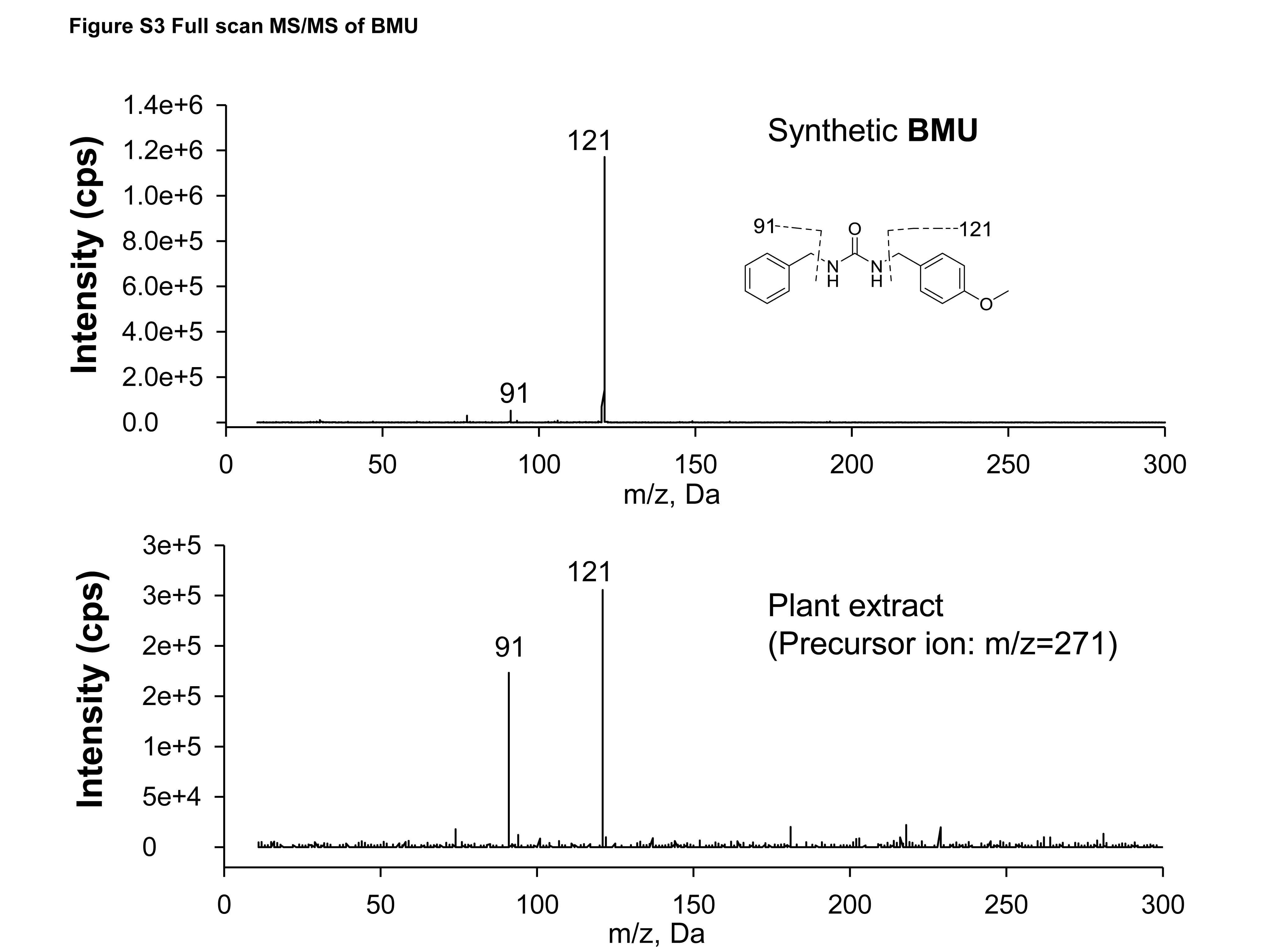

Supplement: S3 Fig — Full scan MS/MS spectra (m/z interval of 10–300) were collected for BMU setting precursor ion m/z as 271 with cone voltage and collision voltage of 35V and 26V, respectively. cps: counts per second. (TIF) [file pone.0117438.s003.tif]

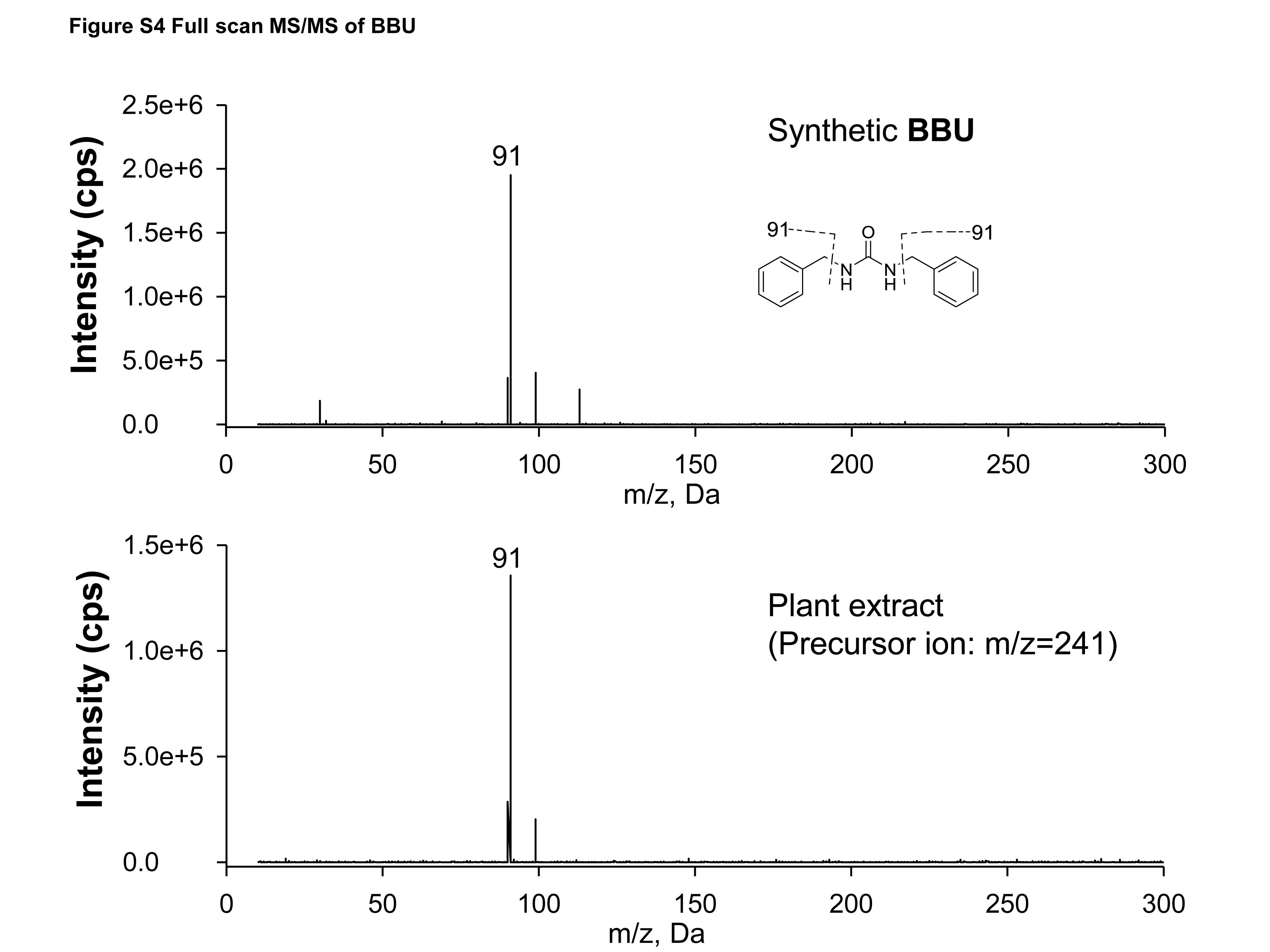

Supplement: S4 Fig — Full scan MS/MS spectra (m/z interval of 10–300) were collected for BBU setting precursor ion m/z as 241 with cone voltage and collision voltage of 35V and 26V, respectively. cps: counts per second. (TIF) [file pone.0117438.s004.tif]

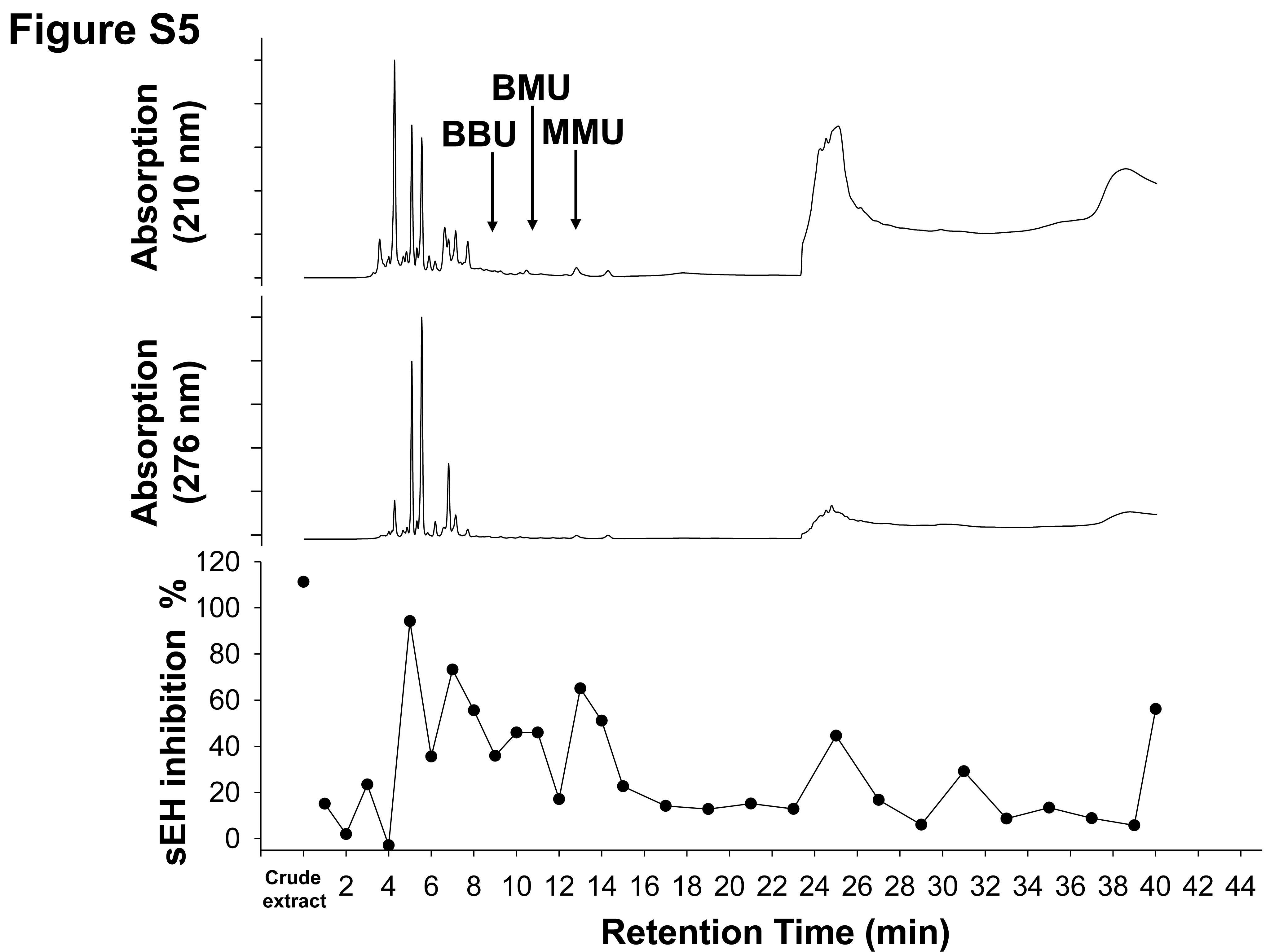

Supplement: S5 Fig — Crude root extract (approximately 2 mg) was injected into a normal phase HPLC column (YMC-Pack SIL-06) and eluted with 20% isopropanol in hexane with a flow rate of 4 ml/min for 15 min, followed by 100% isopropanol with a flow rate of 2 ml/min for 25 min. The relative intensity of the UV absorption at the wavelength of 210 and 276 nm are shown (top and middle). Fractions were collected for every 4 ml of eluent. After the solvent was evaporated the residue was reconstituted in 50 μl DMSO. The inhibition percentage by each fractions (100 times dilution of reconstituted solution) was measured using the CMNPC assay with recombinant human sEH (bottom). The black circles (●) represent the inhibition percentage by each of the fractions. The crude extract mixture (100 times dilution of 2 mg extract/ml DMSO) showed complete inhibition of sEH activity (black circle shown at time 0 min). The retention times of the 3 synthetic ureas (BBU, BMU, and MMU) are indicated by arrows in the figure. (TIF) [file pone.0117438.s005.tif]

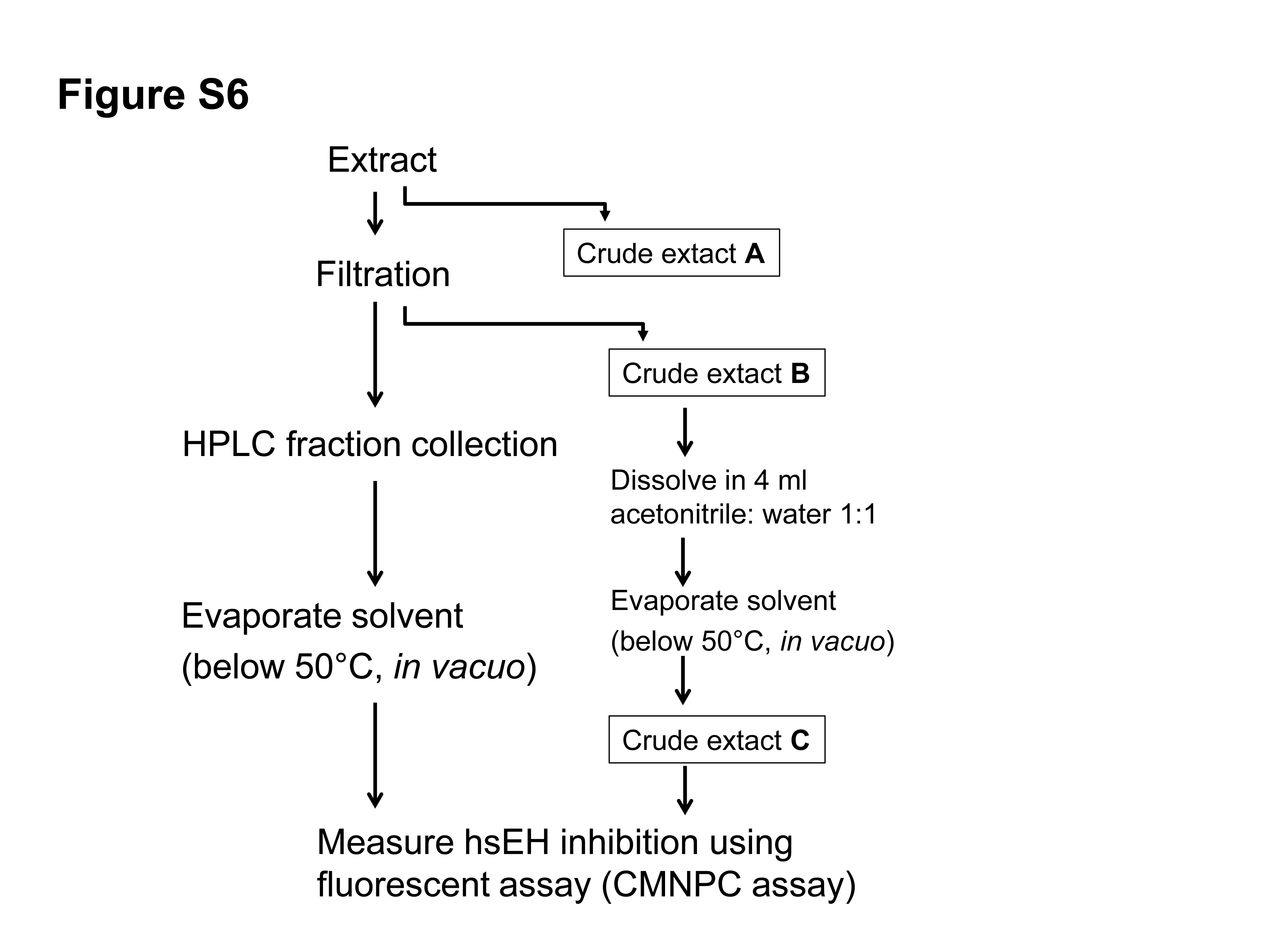

Supplement: S6 Fig — (TIF) [file pone.0117438.s006.tif]

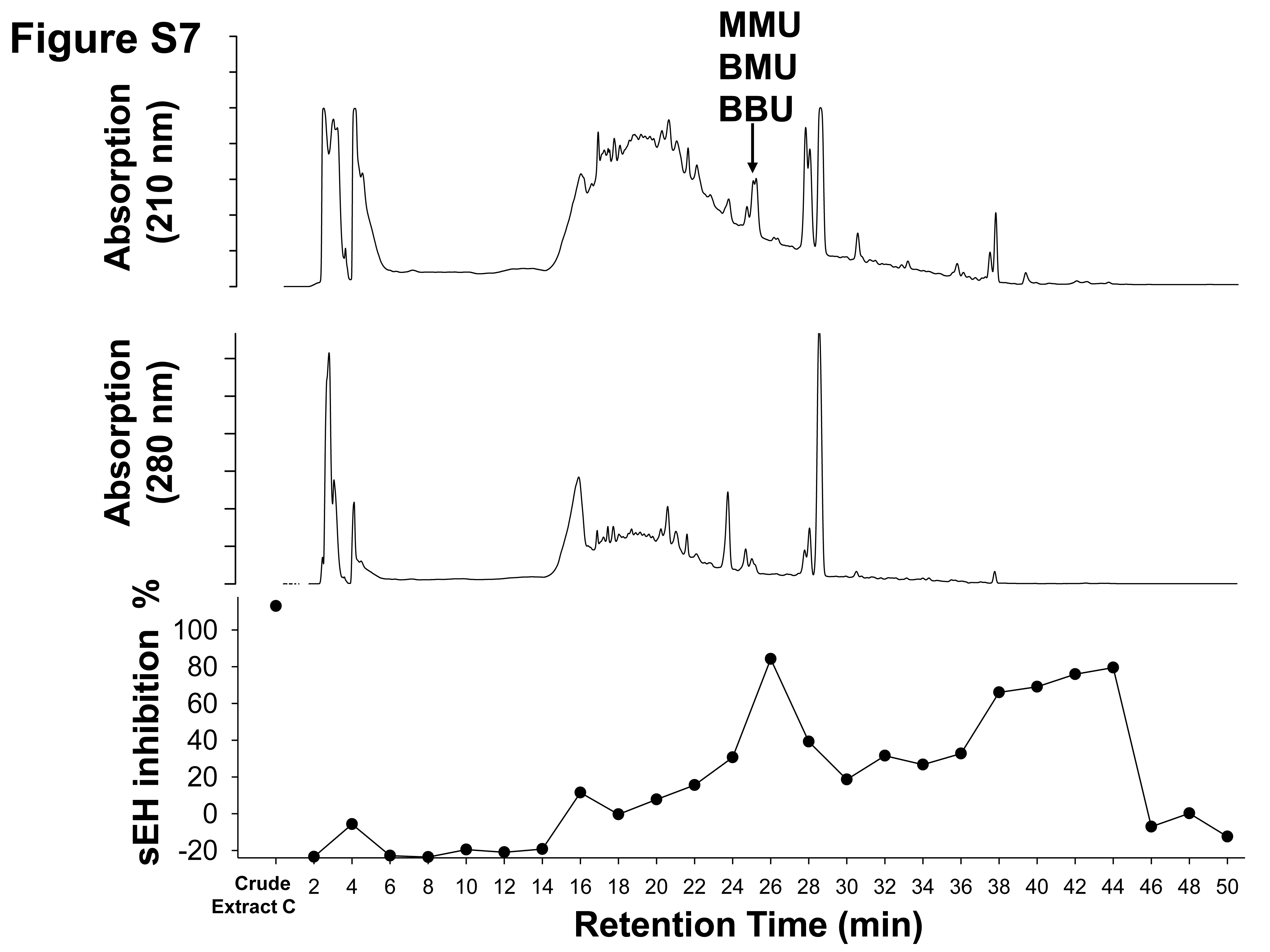

Supplement: S7 Fig — Crude root extract (approximately 5 mg) was injected into a reverse phase HPLC column (Waters SunFire Prep C18, 5 μm, 10x100 mm) and eluted with 10% acetonitrile in water with a flow rate of 2 ml/min for 10 min, followed by a linear gradient elution of acetonitrile 10% to 100% at a flow rate of 2 ml/min for 25 min, and eluted with 100% acetonitrile for 15 min at a flow rate of 2 ml/min. The relative intensity of the UV absorption at the wavelength of 210 and 280 nm are shown (top and middle). The retention times of the 3 synthetic ureas (BBU, BMU, and MMU) are indicated by an arrow in the figure. Fractions were collected for every 4 ml of eluent. After the solvent was evaporated the residue was reconstituted in 50 μl DMSO. The inhibition percentage by each fractions (100 times dilution of reconstituted solution) was measured using the CMNPC assay with recombinant human sEH (bottom). The black circles (●) represent the inhibition percentage by each of the fractions. The crude extract C (100 times dilution of 5 mg extract/ml DMSO) showed complete inhibition of sEH activity (black circle shown at time 0 min). (TIF) [file pone.0117438.s007.tif]
