# Supplementary material for: Potent Natural Soluble Epoxide Hydrolase Inhibitors from Pentadiplandra brazzeana Baillon: Synthesis, Quantification, and Measurement of Biological Activities In Vitro and In Vivo
Source: PLoS One. 2015 Feb 6;10(2):e0117438. doi: 10.1371/journal.pone.0117438 (PMC4319826; doi:10.1371/journal.pone.0117438)
Supplement: S1 Table — (DOCX) [file pone.0117438.s008.docx]

**Table S1. Relative potency of normal phase HPLC fractions**

| HPLC fraction | Relative potency unit^1^ | Recovery %  from crude extract^2^ |
| --- | --- | --- |
| 4-5 min | 0.51 | 11.8 |
| 6-7 min | 0.50 | 11.5 |
| 7-8 min | 0.21 | 4.8 |
| 12-13 min  (contains **MMU**) | 0.33 | 7.7 |
| 13-14 min | 0.18 | 4.2 |
| 39-40 min | 0.18 | 4.1 |
| Total inhibition activity recovered in fractions showing 50% inhibition | 1.91 | 44.1 |
| Crude extract | 4.33 | 100 |

^1^The potency of each of the fractions is presented as a relative potency to the calculated IC_50_ of the extract based on the concentration of **MMU** (Relative potency unit=dilution factor at IC_50_ of each of fraction/dilution factor at calculated IC_50_ based on **MMU**).

^2^The recovery percentage from the crude extract was calculated by the formula: Recovery %=Relative potency unit of the fraction/Relative potency unit of Crude extract× 100.
