# Supplementary material for: Potent Natural Soluble Epoxide Hydrolase Inhibitors from Pentadiplandra brazzeana Baillon: Synthesis, Quantification, and Measurement of Biological Activities In Vitro and In Vivo
Source: PLoS One. 2015 Feb 6;10(2):e0117438. doi: 10.1371/journal.pone.0117438 (PMC4319826; doi:10.1371/journal.pone.0117438)
Supplement: S2 Table — (DOCX) [file pone.0117438.s009.docx]

**Table S2. Relative potency of reverse phase-HPLC fractions**

| HPLC fraction | Relative potency unit^1^ | Recovery % from crude extract **A**^2^ | Recovery % relative to crude extract **C**^3^ |
| --- | --- | --- | --- |
| 22-24 min | 0.05 | 1.5 | 2.7 |
| 24-26 min  (contains **MMU**) | 0.87 | 25.3 | 45.1 |
| 26-28 min | 0.06 | 1.9 | 3.3 |
| 34-36 min | 0.05 | 1.5 | 2.7 |
| 36-38 min | 0.16 | 4.6 | 8.1 |
| 38-40 min | 0.14 | 4.2 | 7.4 |
| 40-42 min | 0.13 | 3.8 | 6.8 |
| 42-44 min | 0.27 | 7.8 | 13.9 |
| Total inhibition activity recovered in fractions above | 1.74 | 50.4 | 90 |
| Crude extract **C**^4^  (filtered & evaporated) | 1.94 | 56.1 | 100 |
| Crude extract **B**^4^  (filtered) | 3.0 | 87 | - |
| Crude extract **A**^4^ | 3.5 | 100 | - |

^1^The potency of each of the fractions is presented as a relative potency to the calculated IC_50_ of the extract based on the concentration of **MMU** (Relative potency unit=dilution factor at IC_50_ of each of fraction/dilution factor at calculated IC_50_ based on **MMU**).

^2^The recovery percentage from the crude extract A was calculated by the formula: Recovery %=Relative potency unit of the fraction/Relative potency unit of Crude extract **A** × 100.

^3^The recovery percentage relative to the crude extract **C** was calculated by the formula: Recovery %=Relative potency unit of the fraction/Relative potency unit of Crude extract **C** (filtered & evaporated) × 100.

^4^Procedure for the preparation of crude extract **A**, **B**, **C** is described in Text S2 and Figure S6.
