# Supplementary material for: Potent Natural Soluble Epoxide Hydrolase Inhibitors from Pentadiplandra brazzeana Baillon: Synthesis, Quantification, and Measurement of Biological Activities In Vitro and In Vivo
Source: PLoS One. 2015 Feb 6;10(2):e0117438. doi: 10.1371/journal.pone.0117438 (PMC4319826; doi:10.1371/journal.pone.0117438)
Supplement: S3 Table — (DOCX) [file pone.0117438.s010.docx]

**Table S3. Effect of extraction solvent on human sEH inhibitory potency**

| Extraction solvent | Crude extract yield^1^  (%weight) | IC_50_^2^  (μg of crude extract/ml) | Relative potency unit^3^ | IC_50_^2^  (μg of root/ml) | Relative inhibitory recovery percentage^4^ |
| --- | --- | --- | --- | --- | --- |
| DCM/MeOH (1:1) | 11 | 13.0 | 4.0 | 118 | 100 |
| Diethylether | 3 | 5.3 | 9.7 | 178 | 66 |
| Ethylacetate | 3 | 5.4 | 9.6 | 180 | 65 |
| Ethanol | 4 | 5.5 | 9.5 | 137 | 86 |

^1^Crude extract was prepared from 100 mg of dried root powder as described in Materials and Methods using the indicated solvent. Crude extract yield (%weight) =weight of crude extract (mg)/weight of dry root sample (100 mg) ×100.

^2^IC_50_ was measured using fluorescent assay using CMNPC as a substrate following the procedure described in Materials and Methods.

^3^The potency of each of the fractions is presented as a relative potency to the calculated IC_50_ of the extract based on the concentration of **MMU** (Relative potency unit=dilution factor at IC_50_ of each of fraction/dilution factor at calculated IC_50_ based on **MMU**).

^4^ Relative inhibitory recovery percentage=IC_50_ (μg of root/ml) of DCM/MeOH (1:1) extract/ IC_50_ (μg of root/ml) of the extract ×100.
