# Supplementary material for: Potent Natural Soluble Epoxide Hydrolase Inhibitors from Pentadiplandra brazzeana Baillon: Synthesis, Quantification, and Measurement of Biological Activities In Vitro and In Vivo
Source: PLoS One. 2015 Feb 6;10(2):e0117438. doi: 10.1371/journal.pone.0117438 (PMC4319826; doi:10.1371/journal.pone.0117438)
Supplement: S1 Text — (DOCX) [file pone.0117438.s011.docx]

**Text S1. Sequence alignments of PCR products from root sample of *P. brazzeana* vs sequences in NCBI database**

**18s rDNA**

Query: NCBI accession number AF070972

Subject 1: Sequence of 18s rDNA from root of *P. brazzeana*

Score Expect Identities Gaps Strand

758 bits(410) 0.0 410/410(100%) 0/410(0%) Plus/Plus

Query 572 GCTCGTAGTTGGACCTTGGGATGGGTCGGCCGGTCCGCCTATGGTGAGCACCGGTCGGCT 631

||||||||||||||||||||||||||||||||||||||||||||||||||||||||||||

Sbjct 1 GCTCGTAGTTGGACCTTGGGATGGGTCGGCCGGTCCGCCTATGGTGAGCACCGGTCGGCT 60

Query 632 CGTCCCTTCTGCCGGCGATACGCTCCTGGCCTTAACTGGCCGGGTCGTGCCTCCGGCGCT 691

||||||||||||||||||||||||||||||||||||||||||||||||||||||||||||

Sbjct 61 CGTCCCTTCTGCCGGCGATACGCTCCTGGCCTTAACTGGCCGGGTCGTGCCTCCGGCGCT 120

Query 692 GTTACTTTGAAGAAATTAGAGTGCTCAAAGCAAGCCTACGCTCTGGATACATTAGCATGG 751

||||||||||||||||||||||||||||||||||||||||||||||||||||||||||||

Sbjct 121 GTTACTTTGAAGAAATTAGAGTGCTCAAAGCAAGCCTACGCTCTGGATACATTAGCATGG 180

Query 752 GATAACATCACAGGATTTCGGTCCTATTGTGTTGGCCTTCGGGATCGGAGTAATGATTAA 811

||||||||||||||||||||||||||||||||||||||||||||||||||||||||||||

Sbjct 181 GATAACATCACAGGATTTCGGTCCTATTGTGTTGGCCTTCGGGATCGGAGTAATGATTAA 240

Query 812 CAGGGACAGTCGGGGGCATTCGTATTTCATAGTCAGAGGTGAAATTCTTGGATTTATGAA 871

||||||||||||||||||||||||||||||||||||||||||||||||||||||||||||

Sbjct 241 CAGGGACAGTCGGGGGCATTCGTATTTCATAGTCAGAGGTGAAATTCTTGGATTTATGAA 300

Query 872 AGACGAACAACTGCGAAAGCATTTGCCAAGGATGTTTTCATTAATCAAGAACGAAAGTTG 931

||||||||||||||||||||||||||||||||||||||||||||||||||||||||||||

Sbjct 301 AGACGAACAACTGCGAAAGCATTTGCCAAGGATGTTTTCATTAATCAAGAACGAAAGTTG 360

Query 932 GGGGCTCGAAGACGATCAGATACCGTCCTAGTCTCAACCATAAACGATGC 981

||||||||||||||||||||||||||||||||||||||||||||||||||

Sbjct 361 GGGGCTCGAAGACGATCAGATACCGTCCTAGTCTCAACCATAAACGATGC 410

**maturase K**

Query: NCBI accession number AY483239

Subject 1: Sequence of maturase K from root of *P. brazzeana*

Score Expect Identities Gaps Strand

843 bits(456) 0.0 458/459(99%) 0/459(0%) Plus/Plus

Query 45 ACACGATTTCCTATACCCACttttttttCGGGAGTATATTTATGTACTTGCTCATGATCA 104

||||||||||||||||||||||||||||||||||||||||||||||||||||||||||||

Sbjct 1 ACACGATTTCCTATACCCACTTTTTTTTCGGGAGTATATTTATGTACTTGCTCATGATCA 60

Query 105 TGGTTTAAATAGATTAAATAGAAACAGATCCATTTTGTTGGAAAATGCGGGTTATGACAA 164

||||||||||||||||||||||||||||||||||||||||||||||||||||||||||||

Sbjct 61 TGGTTTAAATAGATTAAATAGAAACAGATCCATTTTGTTGGAAAATGCGGGTTATGACAA 120

Query 165 GAAATCTAGTTCACTAATTGTGAAACGTTTAATTTTTCGAATGTATGAACAGAATCATTT 224

||||||||||||||||||||||||||||||||||||||||||||||||||||||||||||

Sbjct 121 GAAATCTAGTTCACTAATTGTGAAACGTTTAATTTTTCGAATGTATGAACAGAATCATTT 180

Query 225 GATTATTTCTACTAATGATTTTAACCAAAATCCATTTTTTGGACATAAGAATCATTTGGA 284

||||||||||||||||||||||||||||||||||||||||||||||||||||||||||||

Sbjct 181 GATTATTTCTACTAATGATTTTAACCAAAATCCATTTTTTGGACATAAGAATCATTTGGA 240

Query 285 TTATCAAATGATATCGGCGGTATTTGCAGTGATTGTGGAAATTCCATTTTCCCTAAGATT 344

||||||||||||||||||||| ||||||||||||||||||||||||||||||||||||||

Sbjct 241 TTATCAAATGATATCGGCGGTCTTTGCAGTGATTGTGGAAATTCCATTTTCCCTAAGATT 300

Query 345 AGTATCCTACTTCGAAGGAAAACTACTAGCAAAATCTCATAATTTACAATCAATTCATTC 404

||||||||||||||||||||||||||||||||||||||||||||||||||||||||||||

Sbjct 301 AGTATCCTACTTCGAAGGAAAACTACTAGCAAAATCTCATAATTTACAATCAATTCATTC 360

Query 405 AATATTTCCTTTTTTAGAGGACAAATTCTCACATTTAAATTATGTGTTAGATGTACTAAT 464

||||||||||||||||||||||||||||||||||||||||||||||||||||||||||||

Sbjct 361 AATATTTCCTTTTTTAGAGGACAAATTCTCACATTTAAATTATGTGTTAGATGTACTAAT 420

Query 465 ACCTCACCCCATCCATCTGGAAATCTTGGTTCAAACCCT 503

|||||||||||||||||||||||||||||||||||||||

Sbjct 421 ACCTCACCCCATCCATCTGGAAATCTTGGTTCAAACCCT 459
