# Supplementary material for: Potent Natural Soluble Epoxide Hydrolase Inhibitors from Pentadiplandra brazzeana Baillon: Synthesis, Quantification, and Measurement of Biological Activities In Vitro and In Vivo
Source: PLoS One. 2015 Feb 6;10(2):e0117438. doi: 10.1371/journal.pone.0117438 (PMC4319826; doi:10.1371/journal.pone.0117438)
Supplement: S2 Text — (DOCX) [file pone.0117438.s012.docx]

**Text S2. Methods for HPLC fraction collection** **and sEH inhibition by the fractions**

HPLC fractionation was performed on an Agilent 1200 Series HPLC with a G1322A degasser, a G1311A Quatpump, and a G1315D Agilent detector. For normal phase HPLC, the crude root extract was prepared by the method described in the Materials and Methods from the 2 g of root powder, resulting in 160 mg crude extract. 20 mg of the crude root extract was dissolved into 100 μl of DCM:MeOH (1:1), and 10 μl of the soluble fraction (approximately equivalent to 2 mg of the extract) was injected into a normal phase HPLC column (YMC-Pack SIL-06, 10x300 mm). Analytes were eluted with 20% isopropanol in hexane with a flow rate of 4 ml/min for 15 min, followed by 100% isopropanol with a flow rate of 2 ml/min for 25 min.

For reverse phase HPLC, the procedure is summarized as Figure S6. The crude root extract was prepared by the method described in the Materials and Methods from the 1 g of root powder, resulting in 78 mg crude extract (crude extract **A**). 23 mg of the crude root extract was dissolved into 460 μl of MeOH, and 100 μl of the soluble fraction (crude extract **B**, approximately equivalent to 5 mg of the extract) was injected into a reverse phase HPLC column (Waters SunFire Prep C18 5μm, 10x100 mm). Analytes were eluted with 10% acetonitrile in water with a flow rate of 2 ml/min for 10 min, followed by a linear gradient elution of acetonitrile 10% to 100% at a flow rate of 2 ml/min for 25 min, and eluted with 100% acetonitrile for 15 min at a flow rate of 2 ml/min.

The UV absorption between 190 nm and 400 nm was monitored. After sample injection, 4 ml-fractions were collected. The solvent was evaporated and the residue was reconstituted in 50 μl DMSO. In parallel to these fractions, 100 μl of crude extract **B** was dissolved into 4 ml of acetonitrile:water (1:1), evaporated, and reconstituted in 50 μl DMSO (crude extract **C**).

The inhibitory potency on human sEH was measured using the CMNPC assay as described in the Materials and Methods. Firstly, the inhibition percentage by each fraction (100 times dilution) was measured (Figure S5&S7). Then the inhibitory potency was measured for the fractions showing higher than 50% inhibition and the IC_50_ was determined. The potency of each of the fractions is presented as a relative potency to the calculated IC_50_ of the extract based on the concentration of **MMU** (Relative potency unit=dilution factor at IC_50_ of each of fraction/dilution factor at calculated IC_50_ of the extract based on **MMU**). The reverse phase HPLC fraction collection resulted in the inhibitory recovery of 90% of crude extract **C**. Data in Table S1&S2 show the relative potency of each fraction and the percentage of recovered potency.
